# Identification of a Novel Survival-Related circRNA–miRNA–mRNA Regulatory Network Related to Immune Infiltration in Liver Hepatocellular Carcinoma

**DOI:** 10.3389/fgene.2022.800537

**Published:** 2022-03-02

**Authors:** Yu Gan, Weidan Fang, Yan Zeng, Peijun Wang, Renfeng Shan, Ling Zhang

**Affiliations:** ^1^ Department of Medical Oncology, The First Affiliated Hospital of Nanchang University, Nanchang, China; ^2^ Medical Innovation Center, The First Affiliated Hospital of Nanchang University, Nanchang, China; ^3^ Department of General Surgery, The First Affiliated Hospital of Nanchang University, Nanchang, China; ^4^ Human Genetic Resources Center, The First Affiliated Hospital of Nanchang University, Nanchang, China

**Keywords:** liver hepatocellular carcinoma, competing endogenous RNA network, circular RNAs, bioinformatics, immune infiltration

## Abstract

Increasing studies have reported that circular RNAs (circRNAs) play critical roles in tumorigenesis and cancer progression. However, the underlying regulatory mechanisms of circRNA-related competing endogenous RNA (ceRNA) in liver hepatocellular carcinoma (LIHC) are still unclear. In the present study, we discovered dysregulated circRNAs through Gene Expression Omnibus (GEO) analysis and validated the expression of the top seven circRNAs with upregulated expression by qRT–PCR and Sanger sequencing. Then, the Cancer-Specific CircRNA Database (CSCD) was used to predict the downstream miRNAs of seven circRNAs, and expression and survival analyses through The Cancer Genome Atlas (TCGA) were performed to identify the key miRNA in LIHC. Thereafter, the hsa_circ_0017264-hsa-miR-195–5p subnetwork was successfully constructed. Subsequently, we predicted downstream target genes of hsa-miR-195–5p with TargetScan, miRDB, and mirtarbase and overlapped them with differentially expressed mRNAs to obtain 21 target genes. Gene ontology (GO) and Kyoto Encyclopedia of Genes and Genomes (KEGG) pathway enrichment analyses were performed to predict the biological and functional roles of these target genes. Finally, with Pearson correlation and prognostic value analysis, a survival-related hsa_circ_0017264-hsa-miR-195-5p-CHEK1/CDC25A/FOXK1 axis was established. Gene set enrichment analysis (GSEA) was performed to determine the function of CHEK1/CDC25A/FOXK1 in the ceRNA network. Moreover, immune infiltration analysis revealed that the ceRNA network was markedly associated with the levels of multiple immune cell infiltrates, immune cell biomarkers and immune checkpoints. Overall, the hsa_circ_0017264-hsa-miR-195-5p-CHEK1/CDC25A/FOXK1 network might provide novel insights into the potential mechanisms underlying LIHC onset and progression.

## Introduction

Liver cancer is one of the most common malignant tumors; 75%–85% of liver cancer cases are classified as hepatocellular carcinoma (LIHC), and 10%–15% are classified as intrahepatic cholangiocarcinoma (CHOL) (Sung et al., 2021). The most common risk factors for LIHC are chronic infection with hepatitis B virus (HBV) or hepatitis C virus (HCV), aflatoxin-contaminated foods, heavy alcohol intake, excess body weight, type 2 diabetes, and smoking (Suh et al., 2018). Increasing evidence suggests that the occurrence and development of LIHC are associated with abnormal genetic changes and cancer-related signaling pathways, such as TERT, the WNT/β-catenin pathway, and the mTOR pathway (Li et al., 2020). Currently, due to the lack of available screening methods and noticeable early symptoms, many LIHC patients are diagnosed at an advanced stage, and the treatment outcomes are unsatisfactory (Ogunwobi et al., 2019; Marrero, 2020). Therefore, it is of great significance to explore the molecular mechanisms of LIHC to identify novel diagnostic and prognostic targets.

Circular RNAs (circRNAs) are a type of endogenous noncoding RNA (ncRNA) discovered by Sanger in viroids in 1976 (Sanger et al., 1976). With the development of bioinformatics and high-throughput sequencing technology, circRNAs have been widely observed in viruses, fungi, plants and animals (Wang et al., 2018). Salmena et al. (2011) first proposed the competitive endogenous RNA (ceRNA) hypothesis that circRNAs negatively regulate miRNA expression by interacting with miRNA response elements (MREs) and then regulate intracellular signaling pathways and downstream gene expression. Recently, several studies (Yao et al., 2019; He et al., 2021) have revealed that circRNAs play a pivotal role in the occurrence and development of LIHC through ceRNA networks. In addition, circRNAs can be used as diagnostic or prognostic markers for LIHC patients (Wang et al., 2019; Chen et al., 2020). Hence, we built a circRNA–miRNA–mRNA network to further clarify the detailed mechanism of circRNAs in LIHC.

In this study, we established a survival-related circRNA-miRNA-mRNA ceRNA regulatory network for LIHC. To achieve this goal, we identified differentially expressed circRNAs, miRNAs, and mRNAs in LIHC and predicted the binding relationship between circRNAs, miRNAs and mRNAs through multiple bioinformatic tools. Finally, combined with clinical information from The *Cancer* Genome Atlas (TCGA) database, we successfully constructed a new ceRNA network that is significantly related to the prognosis of LIHC patients. In addition, the Tumor Immune Estimation Resource (TIMER) database was used to estimate the correlation between the ceRNA network and the level of immune infiltration. This study will provide us with a new perspective on the pathogenesis of LIHC and improve the diagnosis and treatment of LIHC.

## Materials and Methods

### Dataset Retrieval

The NCBI Gene Expression Omnibus (GEO) database (http://www.ncbi.nlm.nih.gov/geo/) was used to find potential functional circRNAs in LIHC. Dataset selection was based on the following screening criteria: 1) datasets focused on human LIHC tissue samples; 2) datasets excluded LIHC cell lines or animal LIHC tissue samples; and 3) the number of samples in the selected dataset was more than 10. Hence, we chose the GSE164803 dataset for further research. Next, we obtained miRNA expression profiles, mRNA expression profiles, and clinical information of LIHC patients from the TCGA database (https://portal.gdc.Cancer.gov/). Detailed information on these datasets is shown in Table 1.

**TABLE 1:** Basic characteristics of three datasets in the GEO and TCGA databases.

Data source	Platform	Series	Sample size	
			Tumor	Control
circRNA	GPL19978	GSE164803	6	6
miRNA	TCGA (Cancer Genome Atlas Resea, 2017)	None	50	375
mRNA	TCGA	None	50	374

**Note:** GSE164803 can be accessed here: https://www.ncbi.nlm.nih.gov/geo/query/acc.cgi?acc=GSE164803.

### Differential Expression Analysis of circRNAs, miRNAs and mRNAs

After downloading data from the GEO and TCGA databases, we used Perl (version 5.32.1.1) to analyze and process the dataset. According to the annotation platform file of the expression profile, the probe names were converted to the corresponding gene names, and empty probes were removed. Then, the “limma” package (Ritchie et al., 2015) (version 3.50.0) of R (version 4.1.0)software was used to identify circRNAs, miRNAs and mRNAs that were differentially expressed between tumor tissues and adjacent tissues. Adjusted *p* < 0.05 and |logFoldChange| > 1 were the identification thresholds.

### circBase and Cancer-Specific CircRNA Database Analysis

Circbase (Glažar et al., 2014) (http://www.circbase.org/) is a circRNA database that contains detailed information on circRNAs in humans, mice and many other species. We found the gene sequence of circRNAs through circBase and compared it with the results of Sanger sequencing. CSCD (Xia et al., 2018) (http://gb.whu.edu.cn/CSCD/) is an online tool for studying cancer-specific circRNAs and is used to visualize the circular structure of potential circRNAs and predict the binding relationship between circRNAs and miRNAs.

### Clinical Specimens

Tumor and adjacent normal tissue specimens were collected from 10 HCC patients undergoing surgery at the First Affiliated Hospital of Nanchang University (Nanchang, China). The patients did not receive any radiotherapy or chemotherapy before surgical operation. Normal and tumor tissues from 10 HCC patients were immediately frozen in liquid nitrogen and then stored in −80°C to extract RNA. This study was approved by the Ethics Committee of the First Affiliated Hospital of Nanchang University.

### Ribonucleic Acid Extraction and Quantitative Reverse Transcription–Polymerase Chain Reaction

Total RNA was extracted from the clinical samples and separated using TRIzol (Invitrogen) according to the manufacturer’s protocol. To quantify the expression of circRNAs, miRNAs and mRNAs, cDNA was synthesized using the PrimeScript™ RT reagent Kit (Takara, Dalian, China), and qRT–PCR was performed using TB Green™ Premix Ex Taq II (Takara, Dalian, China) in a Roche LightCycler 96 instrument. The qRT–PCR cycling conditions were as follows: 95°C for 30 s, 40 cycles at 95°C for 5 s, and 60°C for 20 s. The melt curve stage was set as follows: 95°C for 15 s, 60°C for 60 s, and 95°C for 15 s. The primers were designed and synthesized by Sangon Biotech (Shanghai, China). All primer sequences are listed in Table 2. GAPDH served as an endogenous control for circRNAs and mRNAs, and U6 was used as an internal control for miRNA. The relative level of gene expression was calculated using the 2^−ΔCt^ method (Livak and Schmittgen, 2001).

**TABLE 2:** Primer sequences of circRNAs, miRNAs, and mRNAs used for qRT–PCR.

	Forward primer (5′-3′)	Reverse primer (5′-3′)
hsa_circ_0000854	AAG​ACC​ATC​TTA​AGA​GCC​CTG​AAT​CAG	AAG​TCC​GTC​CTG​AGG​TAT​TGG​AG
hsa_circ_0005307	TTC​CTA​GAG​AGA​CCC​TCG​TCC​TT	TCC​TTC​AAT​ATC​TTG​AAG​CTG​GGC​AGA
hsa_circ_0006286	ACT​GCA​GCA​ACT​GCA​GAT​GGA	ATGACGCCTCCCTGTGGG
hsa_circ_0017264	GGA​CAT​GTT​TAC​GGA​AAT​CAA​AGT​TGG	ACT​CCG​AGC​AGC​TTT​GGA​C
hsa_circ_0034049	AGC​AGT​GAT​CCA​TTG​TAT​GTT​CCA​GAT	TCC​TCC​GTT​AAT​CTC​TTC​CAA​CTA​GCA
hsa_circ_0035946	AGT​GCA​CCA​TGT​CCA​TTC​ATA​GTA​GG	TCC​TGC​TAC​AAC​AAA​GTA​GTC​AGC​AAC
hsa_circ_0051488	TCT​CTG​GAA​CAG​CTC​ATC​GCC	TCC​AAA​TGT​GGT​CAG​GAG​GGT​C
CHEK1	GAT​ATG​AAG​CGT​GCC​GTA​GAC​TGT​C	GGA​TAT​TGC​CTT​CTC​TCC​TGT​GAC​C
CDC25A	CTG​ATG​GCA​AGC​GTG​TCA​TTG​TTG	TCA​GGA​CAT​ACA​GCT​CAG​GGT​AGT​G
FOXK1	ACA​CGT​CTG​GAG​GAG​ACA​GC	GAG​AGG​TTG​TGC​CGG​ATA​GA
hsa-miR-195-5p	AGC​AAC​TTT​AGC​AGC​ACA​GAA​A	CAGTGCAGGGTCCGAGGT
GAPDH	ATC​ATC​CCT​GCC​TCT​ACT​GG	TGG​GTG​TCG​CTG​TTG​AAG​TC
U6	CGC​TTC​GGC​AGC​ACA​TAT​AC	TTC​ACG​AAT​TTG​CGT​GTC​ATC

### Sanger Sequencing

CircRNA products amplified with divergent primers, including splicing sites, were validated by Tsingke Biological Technology (Beijing, China).

### Target Gene Prediction and miRNA-mRNA Correlation Analysis

The miRDB (Chen and Wang, 2020) (http://www.mirdb.org/), TargetScan (Agarwal et al., 2015) (http://www.targetscan.org/), and mirtarbase (Chou et al., 2018) (https://mirtarbase.cuhk.edu.cn/∼miRTarBase/miRTarBase_2022/php/index.php) three databases were used to predict target genes of miRNAs. Venny2.1 (https://bioinfogp.cnb.csic.es/tools/venny) was used to compare two groups of molecules to obtain overlapping genes. The starbase (Li et al., 2014) (http://starbase.sysu.edu.cn/) database was used for Pearson correlation analysis between miRNAs and mRNAs.

### Survival and Prognostic Analysis

To evaluate the value of the identified ceRNA network and determine the miRNAs and mRNAs related to prognosis, we downloaded the miRNA and mRNA expression profiles and clinical information of LIHC patients from the TCGA database. The “survival” package in R was used to analyze the prognostic values of miRNAs and mRNAs in LIHC.

### Gene Ontology and Pathway Enrichment Analysis

The “clusterProfiler” (version 4.2.1) and “GOplot” packages (Yu et al., 2012; Walter et al., 2015) of R were used to perform GO annotation and Kyoto Encyclopedia of Genes and Genomes (KEGG) pathway analysis to evaluate the 21 selected mRNAs with prognostic value. *p* < 0.05 served as the cutoff criterion.

### Immune Infiltration Level Analysis of the ceRNA Network

TIMER (Li et al., 2017) is a comprehensive database for investigating the molecular characterization of tumor–immune interactions (https://cistrome.shinyapps.io/timer/). We analyzed the correlation of CHEK1/CDC25A/FOXK1 expression with the abundance of immune infiltrates (B cells, CD4^+^ T cells, CD8^+^ T cells, neutrophils, macrophages, and dendritic cells) via gene modules. Gene expression levels against tumor purity are displayed in the left-most panel. In addition, correlations between CHEK1/CDC25A/FOXK1 expression and gene markers of tumor-infiltrating immune cells were explored via correlation modules.

### Immune Checkpoint Expression Analysis of the ceRNA Network

Gene Expression Profiling Interactive Analysis (GEPIA) (http://gepia.cancer-pku.cn/) is a public database (Tang et al., 2017)) that enables pairwise gene correlation analysis for any given set of TCGA and/or GTEx expression data. The “correlation analysis” module was used to assess the correlation between the expression of genes in the ceRNA regulatory network and the expression of immune checkpoints.

### Statistical Analysis

The qRT–PCR results were statistically analyzed using GraphPad Prism 7. Paired Student’s t-test was used to evaluate the differences in circRNAs, miRNA and mRNAs expression between the cancer group and the control group. *p* < 0.05 was considered to indicate a statistically significant difference.

## Results

### Prediction of the Differentially Expressed circRNAs in LIHC

**FIGURE 1:**
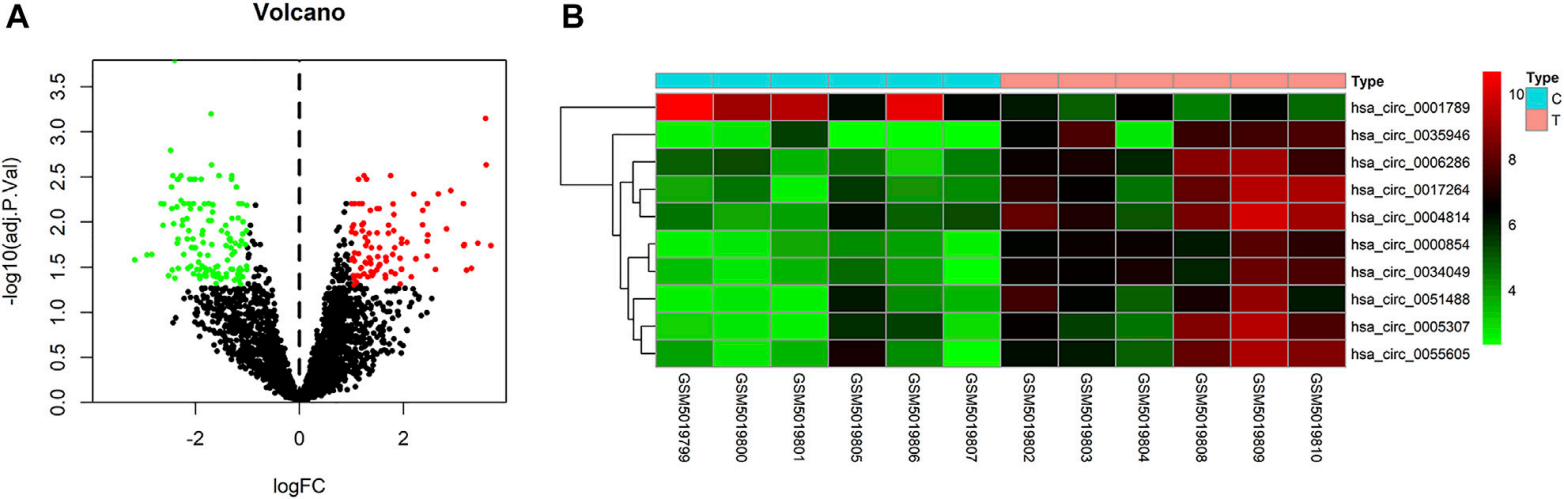
Differentially expressed circRNAs in GSE164803. **(A)** Volcano plot of differentially expressed circRNAs in LIHC from the GSE164803 dataset. The red dots and green dots represent significantly upregulated circRNAs and downregulated circRNAs, respectively (adjusted *p* < 0.05 and |logFoldChange| > 1). The black dots are circRNAs without significant changes. **(B)** Heatmap of 10 potential circRNAs (|logFoldChange| > 3). The intensity increased from green (relatively lower expression) to red (relatively higher expression). C, control tissues; T, tumor tissues.

**FIGURE 2:**
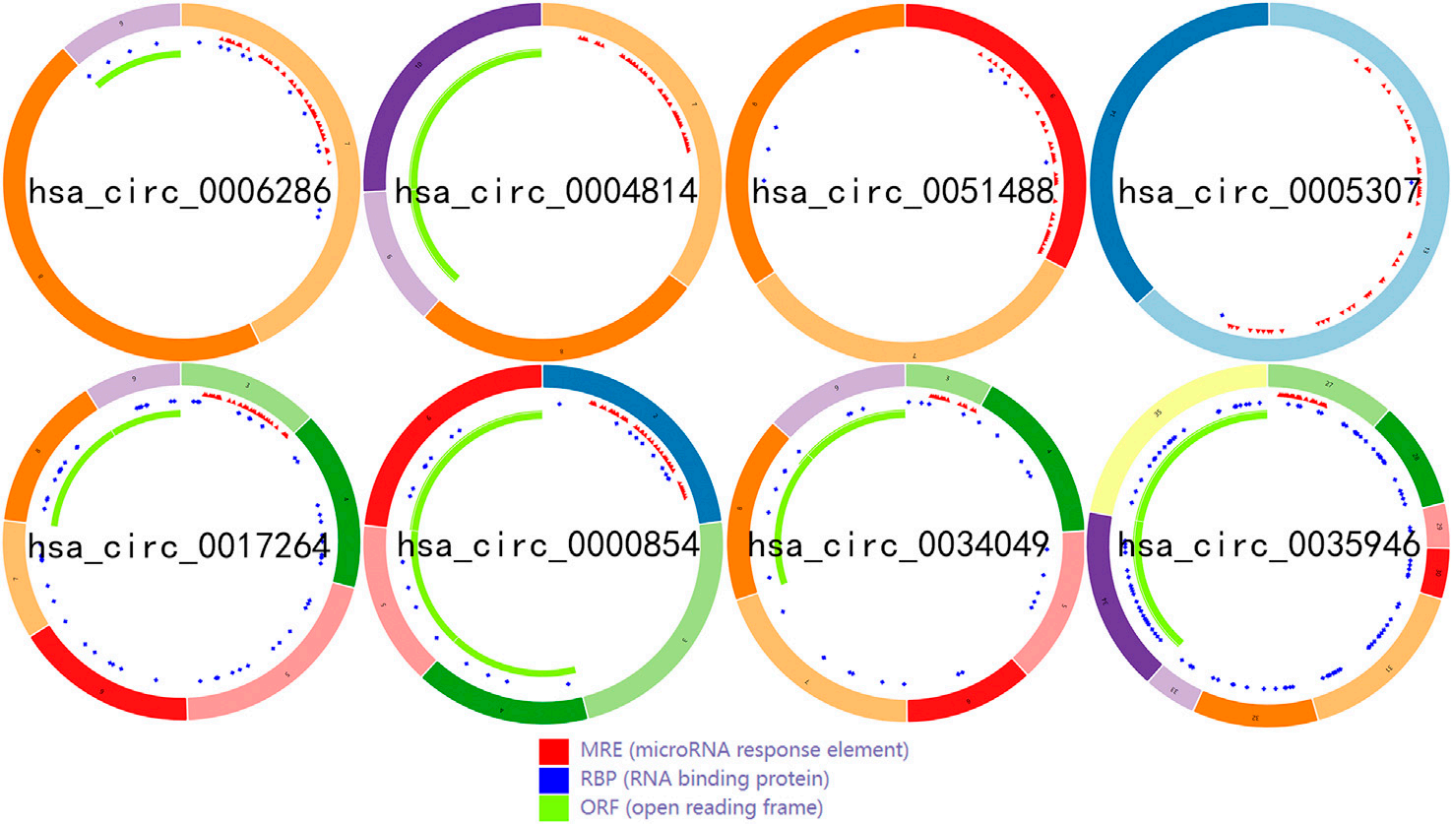
The structural patterns of the top upregulated circRNAs from the CSCD.

To identify some potential circRNAs related to the progression of LIHC, we used the GSE164803 dataset in GEO to analyze the differential expression of liver cancer tissues and adjacent tissues based on the criterion |log2FC| > 1, adjusted *p* < 0.05. The results are displayed in Figure 1A. For more accurate analysis, we changed the screening criteria for differential circRNAs to |log2FC| > 3, adjusted *p* < 0.05. As shown in Figure 1B, 10 circRNAs, including nine upregulated circRNAs and one downregulated circRNA, were identified. The basic information of circRNAs is listed in Table 3. The data included circbase ID, parental gene, genomic length, spliced length, and genome location. In our current work, our goal is to explore prognostic biomarkers specifically increased in LIHC patients, which will be useful in clinical detection and contribute to future drug therapies. We selected nine upregulated circRNAs for further study. Then, we found eight circRNAs structural loop graphs through the CSCD. The graph for hsa_circ_0055605 was not available in the database, but the others are shown in Figure 2. It can be seen that these circRNAs all have miRNA response elements (MREs), which suggests that they may play a role in the progress of LIHC through the ceRNA mechanism.

**TABLE 3:** Basic information for the top 10 differentially expressed circRNAs with a |logFoldChange| > 3.

ID	*p* Value	LogFC	Position	Strand	Genomic length	Spliced length	Gene symbol
hsa_circ_0001789	0.001445	−3.16998	chr8:37734626–37735069	-	443	443	RAB11FIP1
hsa_circ_0006286	8.42E-05	3.152535	chr11:102076623–102080295	+	2672	230	YAP1
hsa_circ_0004814	0.000762725	3.162132	chr2:102033995–102038934	-	4939	430	RFX8
hsa_circ_0051488	0.000722754	3.173776	chr19:45916934–45917292	-	358	141	ERCC1
hsa_circ_0055605	0.002478378	3.21428	chr2:96601201–96610922	-	9721	9721	TCONS_00004336
hsa_circ_0005307	0.00205387	3.309137	chr2:204154487–204157049	+	2562	177	CYP20A1
hsa_circ_0017264	0.000658336	3.431269	chr1:244579282–244587689	-	8407	762	ADSS
hsa_circ_0000854	7.08E-07	3.58042	chr18:60206913–60217693	+	10780	374	ZCCHC2
hsa_circ_0034049	4.12E-06	3.590866	chr15:22835915–22846952	+	11037	681	TUBGCP5
hsa_circ_0035946	0.000754181	3.682886	chr15:66021409–66048810	-	27401	1509	DENND4A

### Verification of the Expression of Candidate circRNAs in LIHC

**FIGURE 3:**
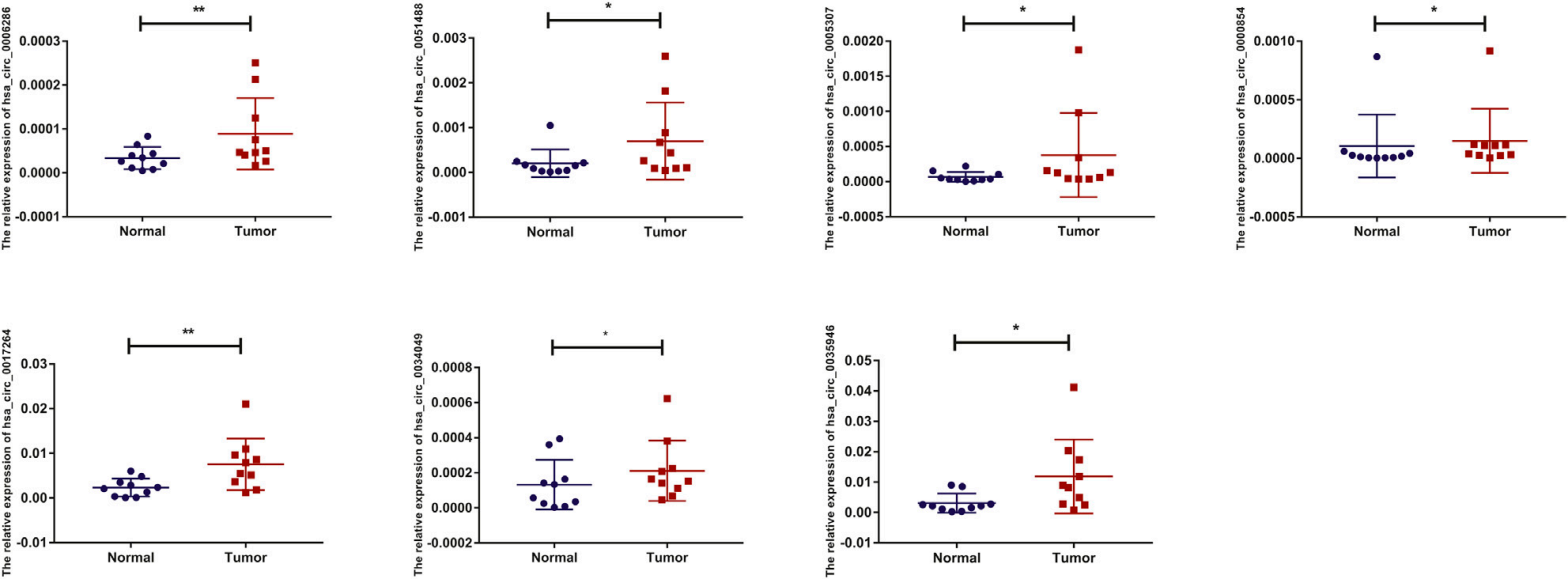
qRT–PCR validation of the expression of candidate circRNAs in LIHC tissues and adjacent normal tissues (*n* = 10).

**FIGURE 4:**
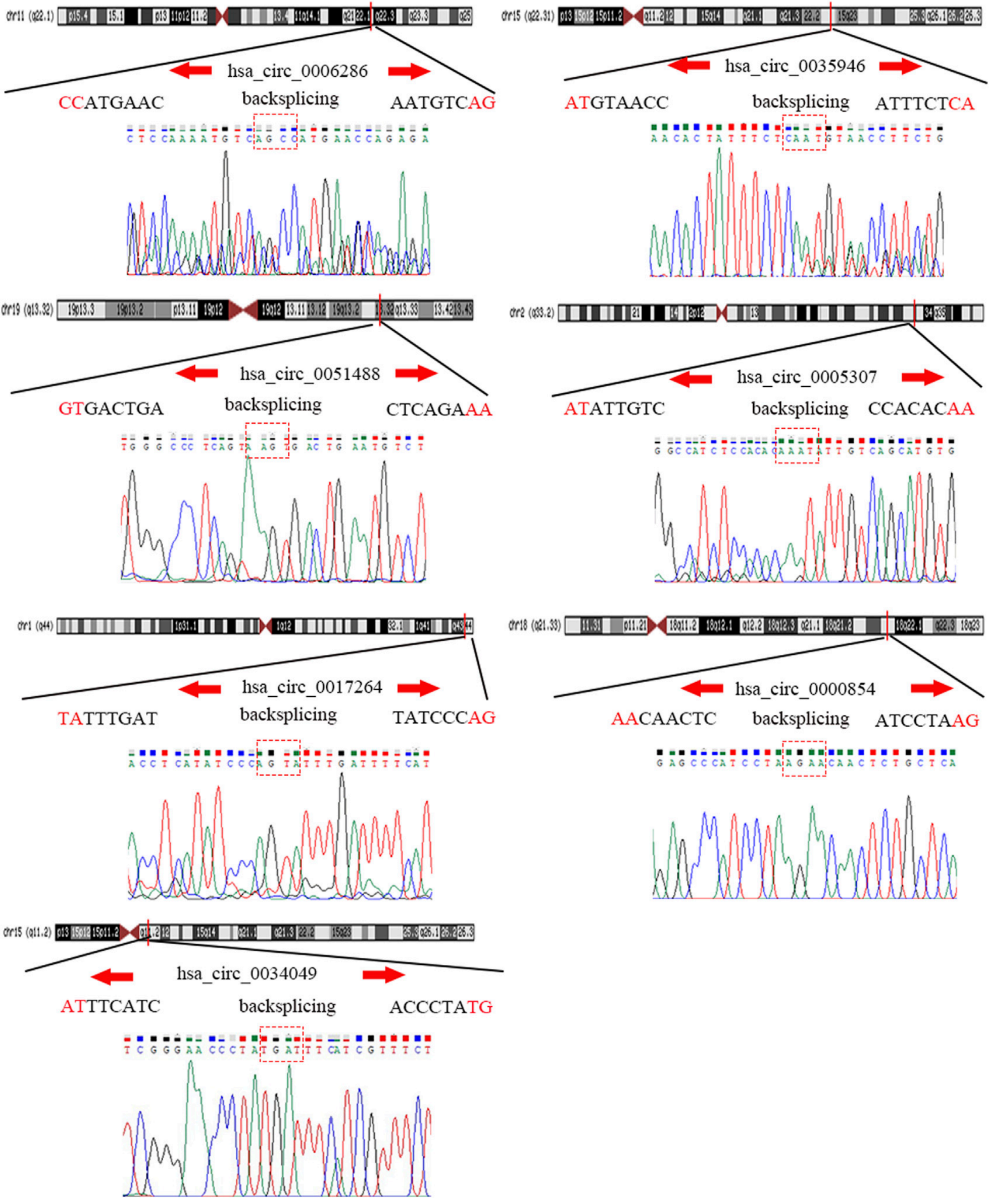
Sanger sequencing verified the specificity of the primers of candidate circRNAs.

To detect and verify the expression of the eight candidate circRNAs, we used divergent primers in clinical specimens. As shown in Figure 3, we found that the expression level of hsa_circ_0004814 was quite low and difficult to detect, and the levels of other circRNAs were consistent with our predictions in the bioinformatics analysis. In addition, we performed Sanger sequencing of the qRT–PCR products to further verify the specificity of these primers, and the results are displayed in Figure 4. Based on this, seven circRNAs (excluding hsa_circ_0004814) were considered the final candidate circRNAs in LIHC.

### Prediction and Analysis of Binding miRNAs of circRNAs in LIHC

**FIGURE 5:**
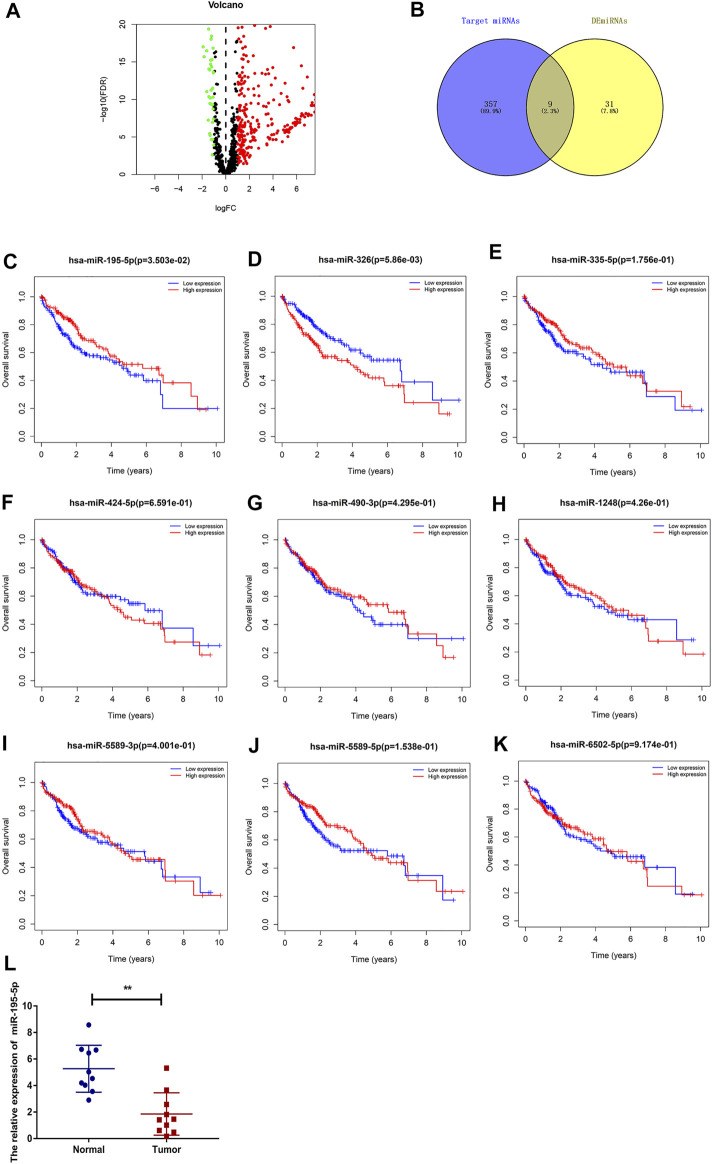
Prediction and analysis of the binding miRNAs of circRNAs in LIHC. **(A)** The volcano plot of DEmiRNAs in LIHC from the TCGA (adjusted *p* < 0.05 and |logFoldChange| > 1). **(B)** Venn diagram analysis of target miRNAs of the seven circRNAs and downregulated DEmiRNAs. **(C–K)** The prognostic value of nine miRNAs in LIHC. **(L)** The expression of hsa-miR-195–5p in LIHC tissues and adjacent normal tissues (*n* = 10).

To predict the binding miRNAs of these circRNAs, we predicted miRNAs that may bind to seven circRNAs through the CSCD database. We found that 366 miRNAs may be targets of the seven circRNAs. According to the ceRNA hypothesis, the expression of circRNAs should be negatively correlated with the expression of miRNAs. Then, we further analyzed the differential expression of miRNAs (DEmiRNAs) in LIHC in the TCGA database. As shown in Figure 5A and Supplementary Figure S1, 260 upregulated and 40 downregulated DEmiRNAs were found. As presented in Figure 5B, we utilized Venn diagrams to illustrate the intersection of the targeted miRNAs and the downregulated DEmiRNAs. Nine miRNAs (hsa-miR-195–5p, hsa-miR-490–3p, hsa-miR-1248, hsa-miR-335–5p, hsa-miR-5589–3p, hsa-miR-6502–5p, hsa-miR-326, hsa-miR-5589–5p, hsa-miR-424–5p) were identified. Moreover, we obtained the clinical information of LIHC patients in the TCGA database and performed survival analysis according to the expression of the nine miRNAs. As shown in Figures 5C–K, we recognized that two miRNAs (hsa-miR-195–5p and hsa-miR-326) were related to prognosis, and only low expression of hsa-miR-195–5p was related to a good prognosis. Besides, as presented in Figure 5L, we further validated the differential expression of hsa-miR-195–5p between the cancer and normal samples.

### Prediction and Analysis of Target Genes of hsa-miR-195-5p in LIHC

**FIGURE 6:**
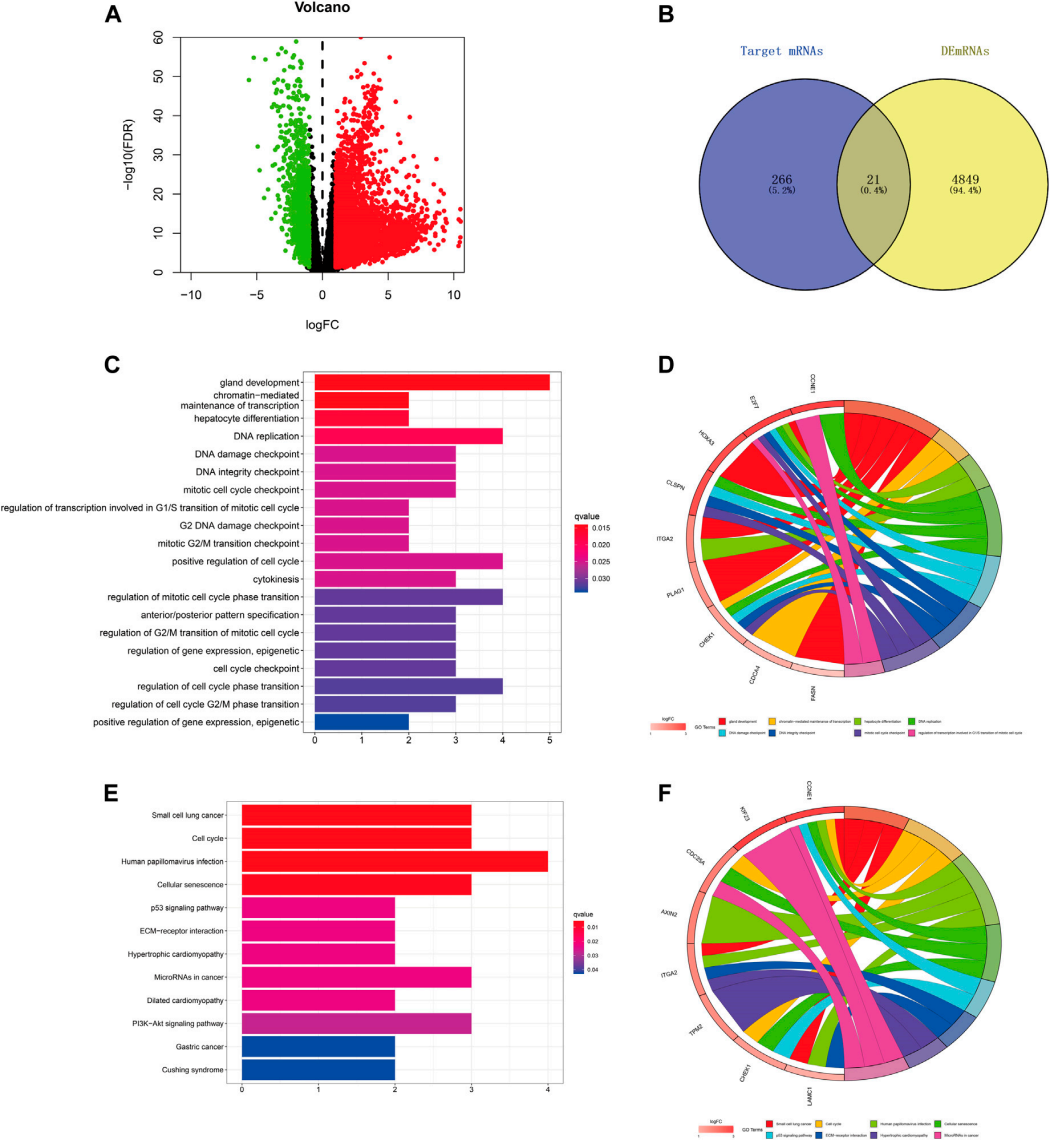
Prediction and analysis of target genes of hsa-miR-195–5p in LIHC. **(A)** The volcano plot of differentially expressed mRNAs in LIHC from TCGA (adjusted *p* < 0.05 and |logFoldChange| > 1). **(B)** Venn diagram analysis of the target mRNAs of hsa-miR-195–5p and upregulated DEmRNAs. **(C–F)** GO and KEGG pathway analyses of the 21 mRNAs.

We predicted the target genes of hsa-miR-195–5p via three databases (miRDB, mirtarbase, and TargetScan). As shown in Figures 6A,B and Supplementary Figure S2, a total of 21 mRNAs (CCNE1, CDCA4, ITGA2, E2F7, CBX2, KIF23, CDC25A, CHEK1, RASEF, HOXA10, PHF19, AXIN2, PLAG1, OSBPL3, CEP55, CLSPN, TPM2, FASN, HOXA3, LAMC1, FOXK1) were identified from the intersection of target genes and upregulated differentially expressed mRNAs (DEmRNAs) of LIHC in the TCGA database. Then, we applied GO and KEGG pathway enrichment analyses to clarify the potential biological processes and signaling pathways of the 21 target genes. As shown in Figures 6C–F, GO analysis revealed that the 21 target genes were mainly enriched in hepatocyte differentiation, DNA replication, chromatin-mediated maintenance of transcription, positive regulation of cell cycle, DNA damage checkpoint, and DNA integrity checkpoint (adjusted *p* < 0.05). According to the results of the KEGG pathway analysis, the target genes were mainly enriched in the cell cycle, cellular senescence, microRNAs in cancer, the p53 signaling pathway, and the PI3K−Akt signaling pathway (adjusted *p* < 0.05).

### Construction of a Survival-Related hsa_circ_0017264-hsa-miR-195-5p-CHEK1/CDC25A/FOXK1 ceRNA Network in LIHC

**FIGURE 7:**
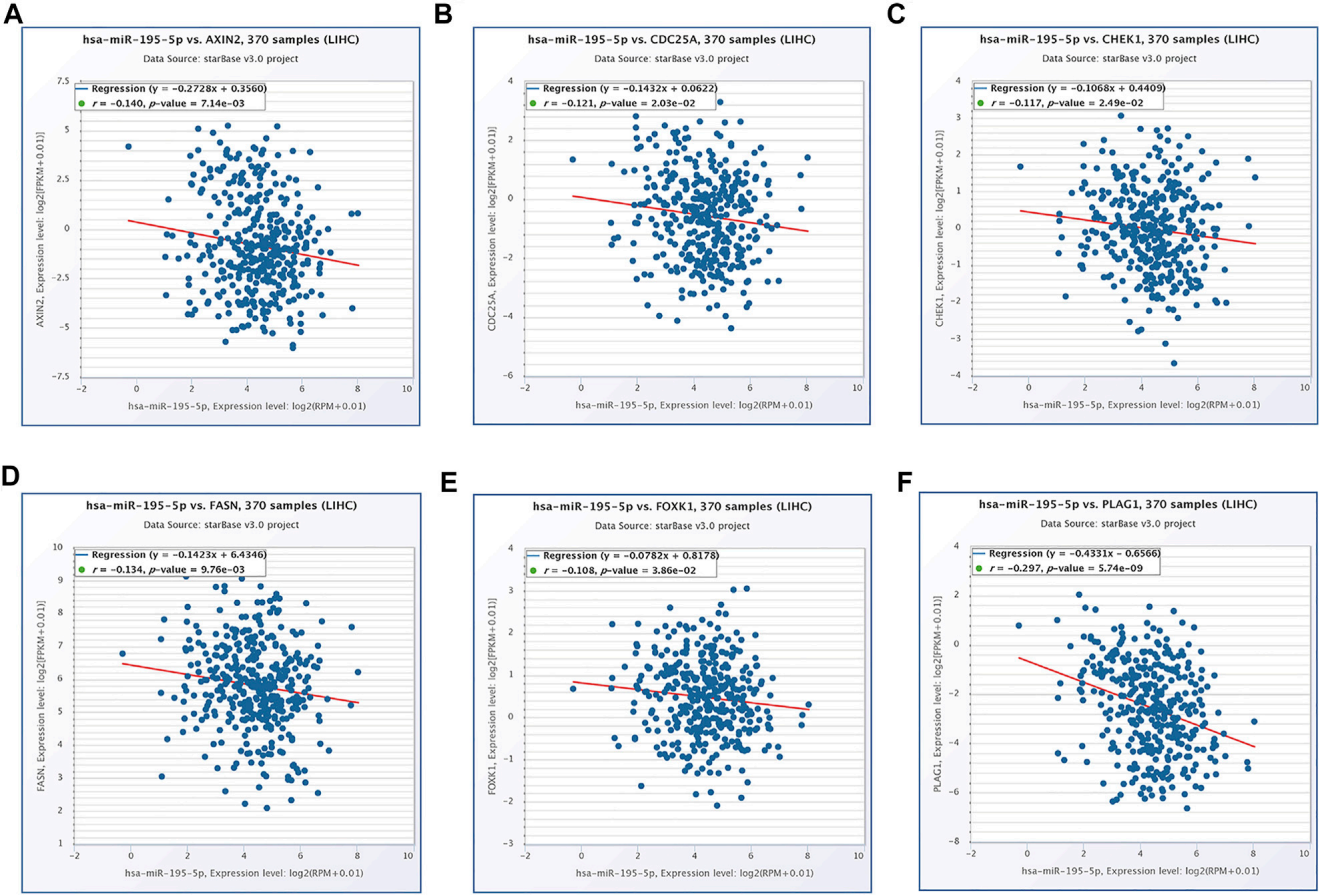
The expression correlation of hsa-miR-195–5p with AXIN2 **(A)**, CDC25A **(B)**, CHEK1 **(C)**, FASN **(D)**, FOXK1 **(E)**, PLAG1 **(F)**.

**FIGURE 8:**
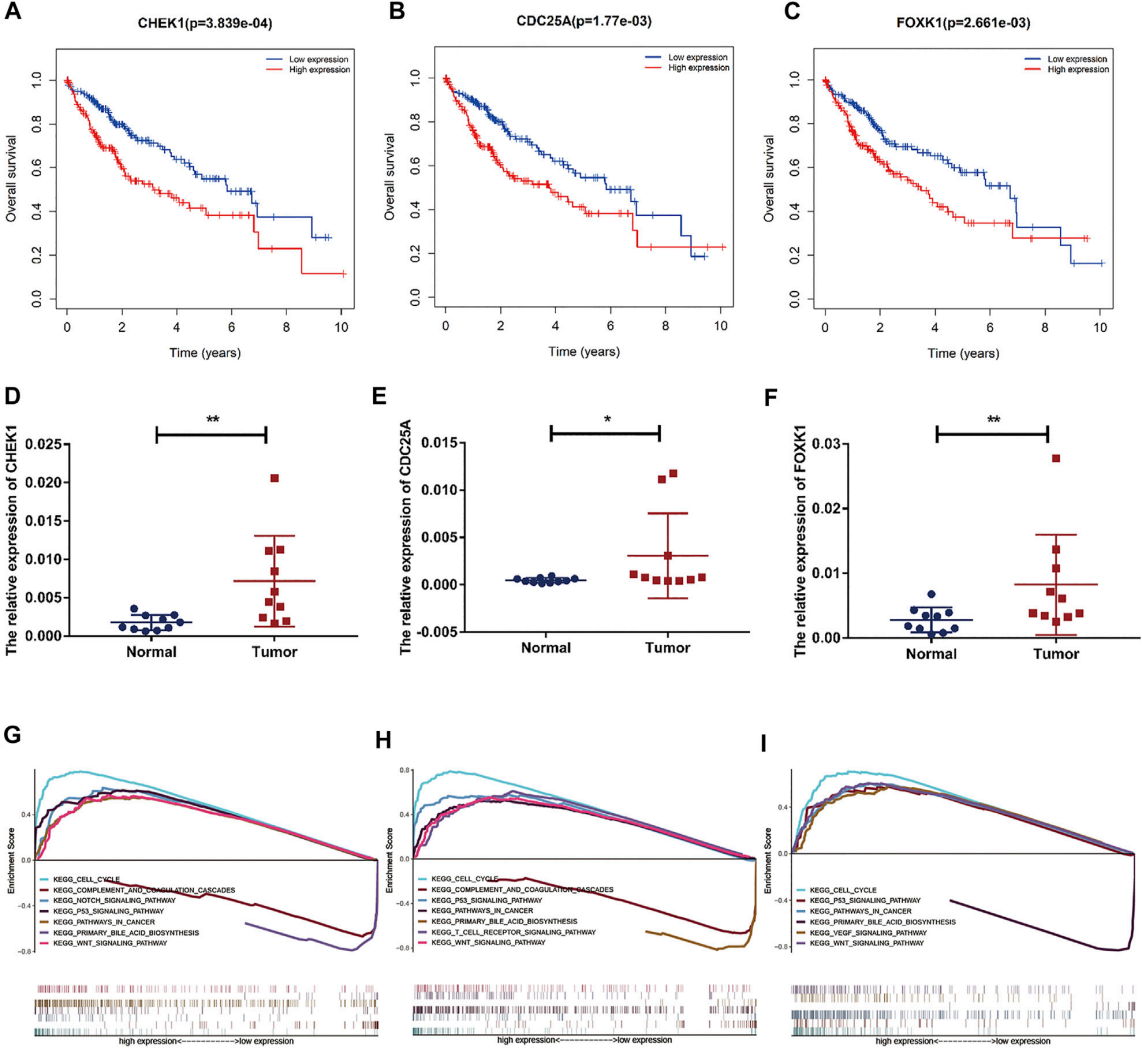
Prognostic assessment and GSEA of target genes. **(A–C)** The prognostic value of CHEK1, CDC25A, FOXK1 in LIHC. **(D–F)** mRNA expression of CHEK1, CDC25A and FOXK1 in LIHC tissues and adjacent normal tissues (*n* = 10) **(G–I)** GSEA of CHEK1, CDC25A and FOXK1 expressed in the ceRNA network.

Based on our above analysis, we found that hsa-miR-195–5p has 21 target genes, which are the intersection of downstream target molecules predicted by three databases and showed significantly upregulated expression in the LIHC-TCGA dataset. As shown in Figures 7A–F, we observed that six target genes had a weak negative correlation with hsa-miR-195–5p but a significant linear relationship. Next, we evaluated the prognostic value of these six mRNAs in LIHC. As presented in Figures 8A–C, among these mRNAs, CHEK1, CDC25A and FOXK1 were negatively correlated with the survival time of patients with LIHC. Moreover, the expression levels of CHEK1, CDC25A and FOXK1 in 10 LIHC patients are presented in Figures 8D–F. Finally, we established a hsa_circ_0017264-hsa-miR-195-5p-CHEK1/CDC25A/FOXK1 ceRNA network, which may provide promising therapeutic targets in treating LIHC in the near future. Then, GSEA was used to study the potential role of CHEK1, CDC25A and FOXK1 in LIHC. As shown in Figures 8G–I, the three genes were positively correlated with the cell cycle, p53 signaling pathway, and WNT signaling pathway and were negatively correlated with primary bile acid biosynthesis.

### Correlations of CHEK1/CDC25A/FOXK1 With Immune Infiltration Level in LIHC

**FIGURE 9:**
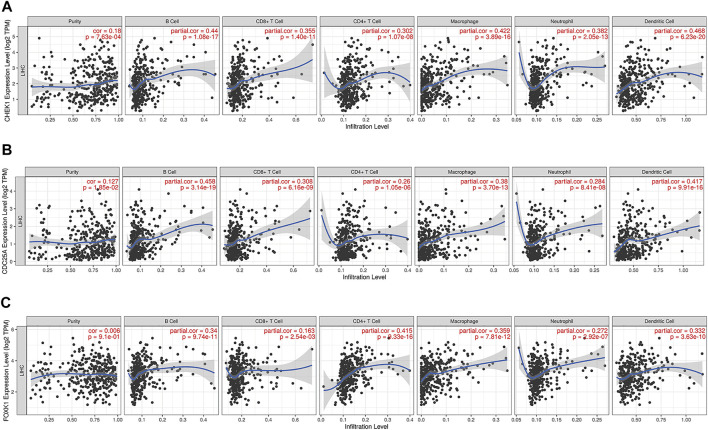
Associations of the expression of CHEK1 **(A)**, CDC25A **(B)**, FOXK1 **(C)** in the ceRNA network with immune infiltration levels in LIHC.

The correlation between CHEK1, CDC25A, FOXK1 and tumor-infiltrating immune cells in the LIHC microenvironment was evaluated by using TIMER. As shown in Figures 9A–C, we found that the expression of genes in the ceRNA regulatory network was significantly related to the infiltration of immune cells (B cells, CD4^+^/CD8^+^ T cells, macrophages, neutrophils and dendritic cells) into the LIHC microenvironment. Furthermore, we explored the relationships between CHEK1/CDC25A/FOXK1 and immune markers by Spearman correlation analysis, and the results are displayed in Table 4.

**TABLE 4:** Analysis of the correlations between the expression of mRNAs in the ceRNA network and that of immune cell biomarkers.

Immune cell	Gene	CHEK1		CDC25A		FOXK1	
		Cor	*p* Value	Cor	*p* Value	Cor	*p* Value
B cell	CD19	0.17	0.001032	0.26	2.00E-07	0.20	7.74E-05
	CD79A	0.06	0.247149	0.17	0.00094	0.09	0.074561
CD8^+^ T cell	CD8A	0.12	0.024253	0.19	0.000269	0.12	0.023008
	CD8B	0.13	0.009814	0.21	3.72E-05	0.06	0.239623
	CD4	0.17	0.001038	0.24	2.02E-06	0.16	0.001694
M1 macrophage	NOS2	0.00	0.973505	-0.09	0.086193	0.10	0.049793
	IRF5	0.39	6.48E-15	0.28	3.27E-08	0.43	1.35E-18
	PTGS2	0.05	0.327301	0.03	0.522283	0.26	3.01E-07
M2 macrophage	CD163	0.07	0.184655	0.10	0.060432	0.16	0.001692
	VSIG4	0.07	0.198693	0.07	0.180497	0.17	0.000892
	MS4A4A	0.07	0.16171	0.08	0.124195	0.18	0.000331
Neutrophil	CEACAM8	0.17	0.000687	0.23	5.95E-06	0.13	0.011642
	ITGAM	0.32	3.33E-10	0.22	1.42E-05	0.35	4.74E-12
	CCR7	-0.02	0.718681	0.05	0.315115	0.13	0.01101
Dendritic cell	HLA-DPB1	0.15	0.003896	0.16	0.001804	0.16	0.001509
	HLA-DQB1	0.14	0.007778	0.16	0.001775	0.15	0.00319
	HLA-DRA	0.14	0.00686	0.12	0.020639	0.20	9.95E-05
	HLA-DPA1	0.10	0.04852	0.08	0.108655	0.24	2.53E-06
	CD1C	0.06	0.23361	0.10	0.050988	0.20	9.81E-05
	NRP1	0.29	6.49E-09	0.16	0.001679	0.45	4.16E-20
	ITGAX	0.27	7.84E-08	0.29	1.23E-08	0.39	5.74E-15

### The Relationships Between CHEK1/CDC25A/FOXK1 and Immune Checkpoints

**FIGURE 10:**
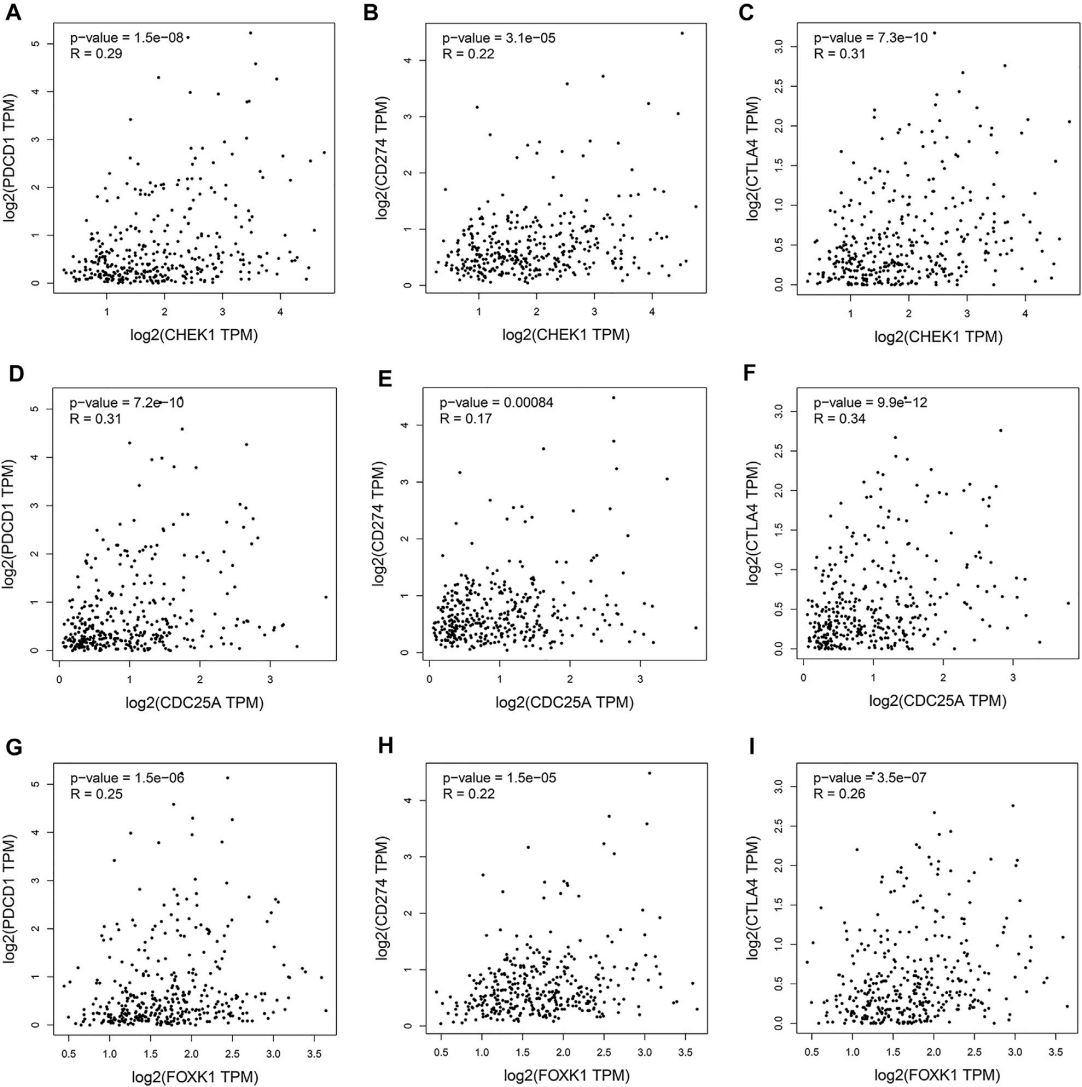
Associations of the expression of CHEK1 **(A–C)**, CDC25A **(D–F)**, FOXK1 **(G–I)** in the ceRNA network with the expression of immune checkpoints in LIHC.

Programmed cell death protein 1 (PD-1), programmed cell death one ligand 1 (PD-L1) and cytotoxic T-lymphocyte-associated protein 4 (CTLA-4) are essential immune checkpoints for tumor immune escape. As shown in Figures 10A–I, the “R” indicates a weak positive correlation among CHEK1, CDC25A, FOXK1 and PD-1, PD-L1, and CTLA-4 in LIHC, with a significant linear relationship. These findings suggest that the hsa_circ_0017264-hsa-miR-195-5p-CHEK1/CDC25A/FOXK1 axis may be involved in facilitating immune escape in the LIHC microenvironment.

## Discussion

In recent years, emerging evidence (Kudo, 2010; Zhang et al., 2020a) has shown that circRNAs are involved in the occurrence and development of tumors through the regulation of parental gene expression, transcriptional translation, and protein modification, but the most crucial role is through the sponge mechanism. Previous research (Huang et al., 2020) indicated that hsa_circRNA_104348 might promote LIHC progression by targeting the miR-187–3p/RTKN2 axis and activating the Wnt/β-catenin pathway. Zhang et al. (2020b) suggested that circTMEM45A sponges miR-665 to promote LIHC progression. In addition, exosomal circUHRF1 (Zhang et al., 2020c) was reported to increase the expression of TIM-3 by sponging miR-449c-5p and inducing NK cell failure. Nevertheless, the molecular characteristics of circRNA-related ceRNA networks in LIHC have not been fully and deeply studied. Therefore, in this study, we constructed a survival-related circRNA-miRNA-mRNA network of LIHC, which will provide potential prognostic biomarkers and therapeutic targets for LIHC.

As a new family of ncRNA molecules, circRNAs affect the occurrence of tumors through glycolysis, metastasis and the cell cycle (Meng et al., 2017). In this study, we performed differential expression analysis on the GSE164803 dataset in the GEO database, identified seven circRNAs with significantly upregulated expression in liver cancer tissues compared with normal liver tissues and verified them in LIHC clinical specimens by qRT–PCR. Among them, one circRNA was previously reported to be closely related to cancer: hsa_circ_0051488 sponges miR-6717–5p, thereby regulating the expression of SATB2, which plays a vital role in lung squamous cell carcinoma (Xiao et al., 2021). However, there is no research focusing on the significance of the other circRNAs, and they are worthy of further study.

**FIGURE 11:**
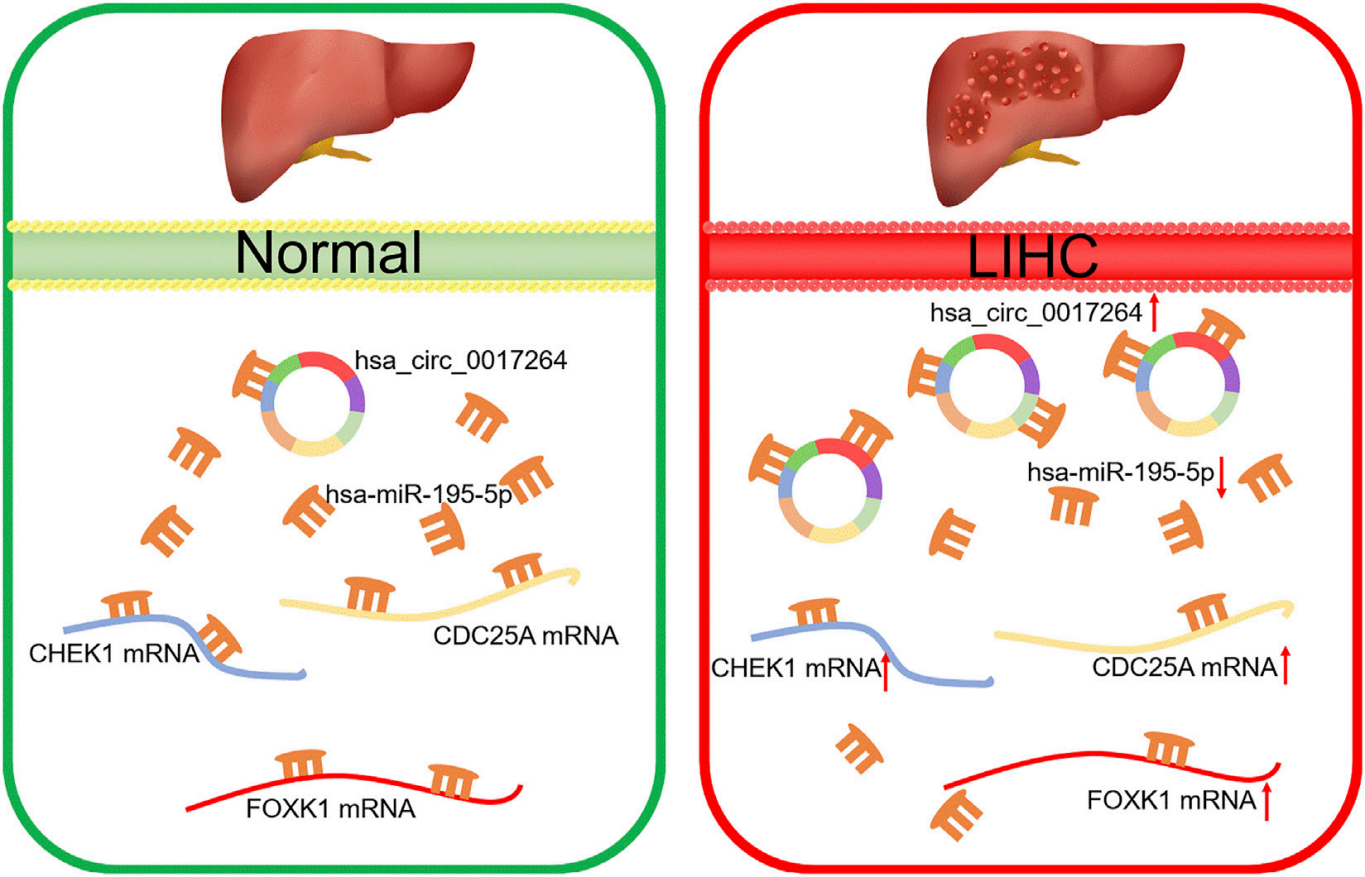
Model of the hsa_circ_0017264-hsa-miR-195-5p-CHEK1/CDC25A/FOXK1 axis in LIHC.

MiRNAs, a class of small molecules 21–25 nucleotides in length, are closely related to tumor occurrence, development, metastasis, and drug resistance (Santarpia et al., 2013; Bottai et al., 2014). In the present study, nine DEmiRNAs were identified in the first step. Then, to enhance the reliability of our research, we conducted prognostic analysis on these DEmiRNAs and finally selected hsa-miR-195–5p for further investigation. Studies have shown (Xu et al., 2015) that miR-195–5p inhibits the proliferation, migration and invasion of liver cancer cells by regulating PHF19. Furthermore, Cai et al. (2018) found that PRR11 is an important downstream mediator of the suppressive effects of miR-195 on prostate cancer progression. These investigations demonstrated that hsa-miR-195–5p plays a tumor suppressor role in various cancers, which partially increased the reliability of our results. To understand the downstream mechanism of hsa-miR-195–5p, we predicted its target genes by employing miRDB, mirtarbase, and TargetScan. Then, we overlapped these target genes with DEmRNAs, and 21 intersecting mRNAs were obtained. GO and KEGG analyses revealed that the 21 genes played a crucial role in many cancer-related biological functions and pathways. Next, we performed prognostic analysis and correlation analysis for the 21 mRNAs and screened the three mRNAs to establish the circRNA-miRNA-mRNA network (Figure 11). Moreover, CHEK1 (Bao et al., 2018; Gong et al., 2020), CDC25A (Liu et al., 2012; Liu et al., 2021), and FOXK1 (Chen et al., 2021; Meng et al., 2021) have been reported as oncogenes, which is consistent with our previous analysis. CHEK1 (Stelzer et al., 2016) is a serine/threonine-specific protein kinase that mediates cell cycle arrest when DNA damage occurs. It was reported (Wu et al., 2019) that CHEK1 overexpression leads to the development of human malignant tumors. As one of the cell cycle regulators, CDC25A participates in the radioresistance of various tumor cells (Ding et al., 2019)). FOXK1 (Feng et al., 2021) is a member of the FOX transcription factor family. The dysregulation of FOXK1 expression and subcellular localization leads to the uncontrolled development and progression of many tumors. Furthermore, GSEA revealed that CHEK1, CDC25A and FOXK1 were enriched in the cell cycle, pathways in cancer, and the P53 signaling pathway, which are correlated with cancer pathogenesis.

Accumulating studies (Jiang et al., 2021; Zhou et al., 2021) have found that tumor-infiltrating immune cells, including B cells, CD4+/CD8+ T cells, macrophages, neutrophils and dendritic cells, affect the efficacy of chemotherapy and immunotherapy and thus affect the prognosis of patients. Our work showed that the ceRNA regulatory network was significantly related to the infiltration of immune cells and the expression of immune checkpoints. Based on the above research, we speculated that the ceRNA regulatory network discovered in this study may be involved in the immune escape of tumor cells. The results will help improve the efficacy of immunotherapy for LIHC.

However, this study has some limitations that need to be considered. First, the construction of a circRNA–miRNA–mRNA regulatory network mainly relies on a series of bioinformatic analyses and databases, and its authenticity and accuracy need to be verified in more experiments. Second, we could not obtain all the clinicopathological data for each LIHC patient in the TCGA database, so the survival analysis may have lower statistical power. Finally, the sample size of the analyzed dataset from the GEO and TCGA was not large, and the number of paired samples was small.

In conclusion, we discovered the survival-related hsa_circ_0017264-hsa-miR-195-5p-CHEK1/CDC25A/FOXK1 regulatory network through a series of bioinformatics analyses. Furthermore, this regulatory network can alter the effect of immunotherapy by affecting tumor immune cell infiltration and immune checkpoint expression, which provides new therapeutic targets for clinical LIHC treatment.

## Data Availability

The original contributions presented in the study are included in the article/Supplementary Material, further inquiries can be directed to the corresponding author.
