# Supplementary material for: Identification of a Novel Survival-Related circRNA–miRNA–mRNA Regulatory Network Related to Immune Infiltration in Liver Hepatocellular Carcinoma
Source: Front Genet. 2022 Mar 2;13:800537. doi: 10.3389/fgene.2022.800537 (PMC8924452; doi:10.3389/fgene.2022.800537)
Supplement: Supplementary file 1 [file Image2.pdf]

Supplementary Material

1 Supplementary Figures

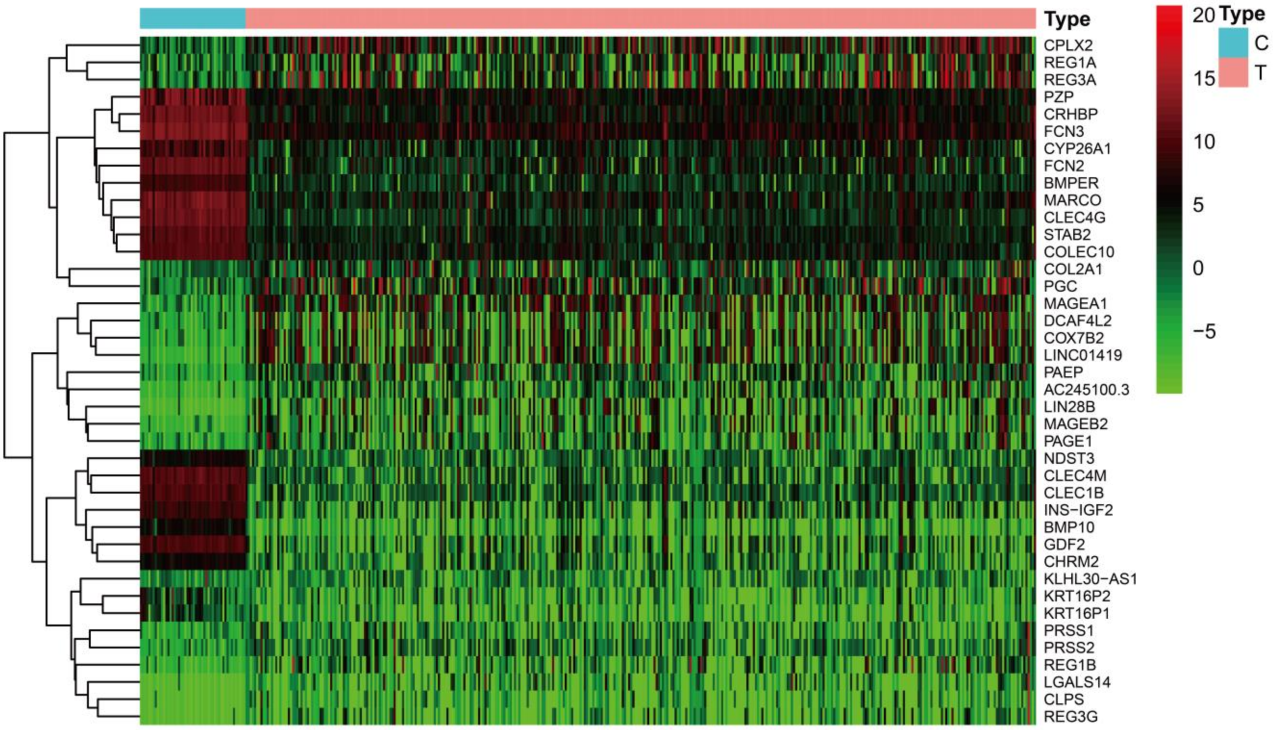

Supplementary Figure 2: Heat map of the 20 most upregulated and downregulated differential expression of mRNAs (DEmRNAs). The intensity increased from green (relatively lower expression) to red (relatively higher expression). C, control tissues; T, tumor tissues.
