# Supplementary material for: Identification of a Novel Survival-Related circRNA–miRNA–mRNA Regulatory Network Related to Immune Infiltration in Liver Hepatocellular Carcinoma
Source: Front Genet. 2022 Mar 2;13:800537. doi: 10.3389/fgene.2022.800537 (PMC8924452; doi:10.3389/fgene.2022.800537)
Supplement: Supplementary file 2 [file Image1.pdf]

Supplementary Material

1 Supplementary Figures

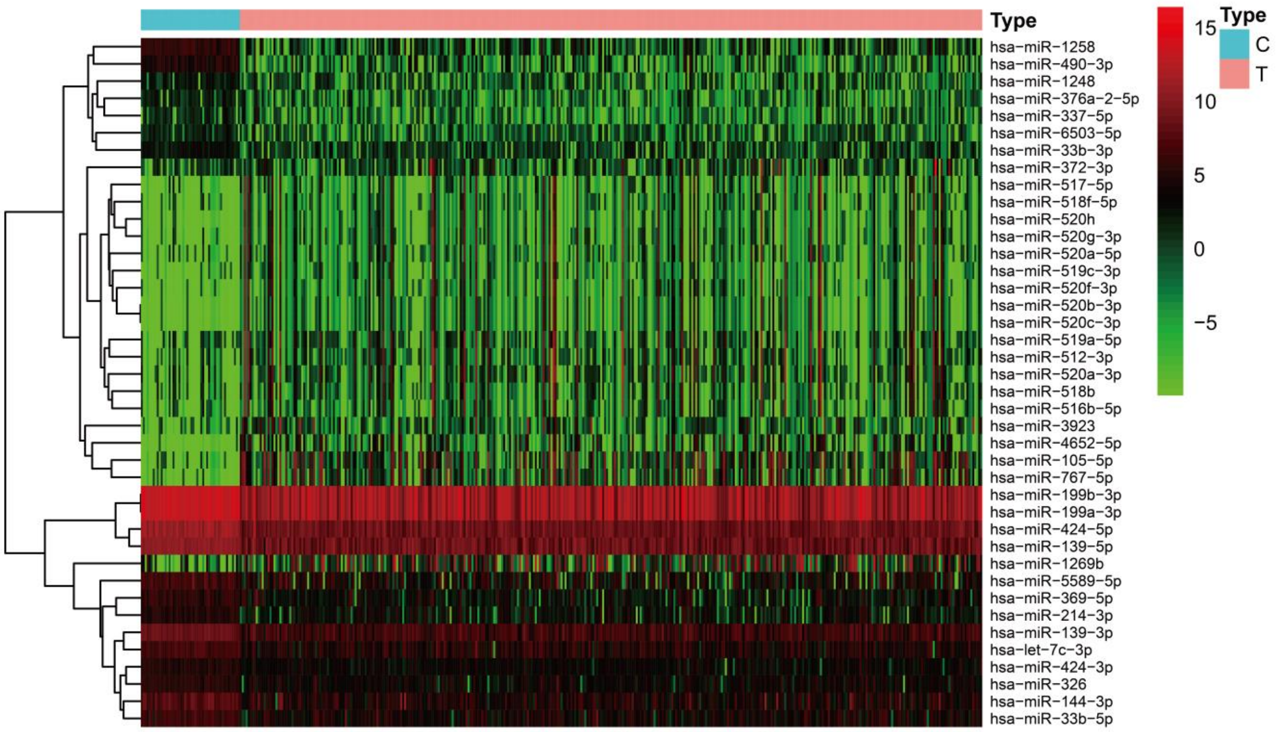

Supplementary Figure 1. Heat map of the top 20 upregulated and downregulated differential expression of miRNAs (DEmiRNAs). The intensity increased from green (relatively lower expression) to red (relatively higher expression). C, control tissues; T, tumor tissues.
